# 2437: Determinants of depression among women from a large community engagement project

**DOI:** 10.1017/cts.2017.107

**Published:** 2018-05-10

**Authors:** Deepthi S. Varma, Jasmine Mack, Linda Cottler

**Affiliations:** Clinical and Translational Science Institute, University of Florida, Gainesville, FL, USA

## Abstract

OBJECTIVES/SPECIFIC AIMS: Depression is one of the leading causes of diseases and disability among women of all ages in the United States. Lack of resources to meet one’s daily needs, access to health care, job opportunities, and drug use significantly contribute to depression among women. This paper aimed to explore the determinants of depression among women from a large community-based sample. METHODS/STUDY POPULATION: HealthStreet is a community engagement research initiative at the University of Florida that utilizes the community health worker (CHW) model to assess health concerns and conditions of community members and link them to available social and medical services and health research. From October 2011 through December 2016, CHWs assessed 8469 community members from various locations in the community such as grocery stores, bus stops, health fairs, laundromats, and others. Among these 8469 participants contacted and assessed by the CHWs, 4952 (58.5%) were women. RESULTS/ANTICIPATED RESULTS: Of the total 8469 participants, 4952 were women and 1839 (37.1%) reported ever having depression. Mean age of women who reported depression was 44.1 years (SD±14.4). Women who were current users of 3 or more drugs were 10 times more likely (95% CI: 5.73, 18.40; OR 10.27) to report depression compared with those who did not currently use any drugs. Those who were food insecure in the past 12 months (95% CI: 1.970, 2.576; OR 2.253) were twice more likely to report depression, while never married (95% CI: 0.576, 0.771; OR 0.666), and currently unemployed (95% CI: 0.535, 0.715; OR 0.619) women were less likely to report depression. Chronic health conditions such as hypertension (41.6% vs. 33.7%), diabetes (14% vs. 10.5%), and cancer (12.1% vs. 8.3%), and comorbid psychiatric symptoms such as anxiety (54.2% vs. 10.8%) and bipolar disorder (23.8% vs. 2.8%) were significantly higher (*p*<0.001) among women with depression compared with their counterparts. Significantly more women without a history of depression had medical insurance (68.8% vs. 64.3%) as compared with women with depression. DISCUSSION/SIGNIFICANCE OF IMPACT: Depression was associated with food insecurity and drug use. The impact of drug use continues to be a major mental health concern among community-based women. Further, these findings emphasize the importance of community engagement programs such as HealthStreet, which utilizes the CHWs’ model to link community members to social and medical services within the community, in improving the mental health of women.

